# Imaging of the skin microvascularization using spatially depolarized dynamic speckle

**DOI:** 10.1117/1.JBO.27.4.046003

**Published:** 2022-04-27

**Authors:** Elise Colin, Aurélien Plyer, Muriel Golzio, Nicolas Meyer, Gilles Favre, Xavier Orlik

**Affiliations:** a Paris Saclay University, DTIS, ONERA, Palaiseau, France; b ITAE Medical Research, Pechabou, France; c CNRS IPBS, UMR 5089, Toulouse, France; d IUC et CHU de Toulouse, Toulouse, France; e Centre de Recherches en Cancérologie de Toulouse, Inserm UMR1037, CNRS UMR5071, Université Toulouse 3, Toulouse, France; f Institut Universitaire du Cancer de Toulouse-Oncopole, Institut Claudius Regaud, Toulouse, France; g Toulouse University, ONERA/DOTA, Toulouse, France

**Keywords:** dynamic speckle, polarization, coherent imaging, laser, skin, dermatology

## Abstract

**Significance:**

We propose a technique devoted to real-time high-resolution imaging of skin microvascularization.

**Aim:**

The process utilizes the temporal variation of the spatially depolarized optical speckle field generated by moving red blood cells when illuminated with fully polarized coherent light.

**Approach:**

Polarimetric filtering prevents the contribution of surface scattering from reaching the camera and thus favors the detection of multiscattered photons from the deeper layers of the skin.

**Results:**

Full-field images reveal the microvasculature with a spatial resolution of 80  μm. The acquisition speed allows for real-time applications.

**Conclusions:**

We demonstrate the ability of this method to determine in 1 s a stable and reliable microvascular activity, enabling numerous clinical applications that require quantitative measurements.

## 1 Introduction

Human microvascularization provides nutrient and waste exchange at the cellular level. It exhibits a remarkable potential to modulate blood flow inside microvessels to adapt permanently to metabolic needs. However, we know that this capacity is altered in many diseases, such as skin cancer,^1^ diabetes,^2^^,^^3^ hypertension,^4^ Alzheimer’s disease,^5^ and even very recently SARS-COV-2.^6^ Thus, we expect *in vivo* observation of the human skin microvascularization to allow for early and reliable diagnosis. Moreover, a real-time monitoring ability could broaden the application field to flap surgery and early detection of sepsis.^7^ However, despite the strong therapeutic potential that could result in such observations, a real-time noninvasive, high-resolution imaging technique to visualize the skin microvascularization of the whole body remains unfulfilled. Among the main existing techniques for *in vivo* imaging of the vasculature, ultra-high resolution computed tomography angiography has recently reached the spatial resolution of 0.2 mm,^8^ but it requires ionizing radiation and contrast agents. Magnetic resonance angiography (MRA), eventually optimized with contrast agents, is typically limited to a millimeter spatial resolution.^9^ Photoacoustic imaging uses an optical excitation by light pulses having energy that is converted into an acoustic wave by moving scatterers. The principle relies on acoustic detection and thus limits the spatial resolution to some hundreds of microns.^10^ Super-resolution ultrasound imaging^11^^–^^14^ has allowed for overcoming the acoustic diffraction limit using gas-filled microbubbles providing a high echogenic contrast agent. It exhibits one of the best spatial resolutions, with a value of 40  μm being recently demonstrated,^14^ and an achievable penetration depth of 10 mm,^12^ but the use of a contrast agent is inherent to the technique and is invasive. In general, purely optical imaging techniques benefit from short wavelengths that favor better spatial resolution but at the expense of smaller penetration depths. Among these techniques, optical coherence tomography^15^ is a depth resolved scanning imaging modality that uses low coherence interferometry. It benefits from a spatial resolution on the order of few microns and is typically limited to submillimeter penetration depth^16^^,^^17^ due to the inherent use of a wide optical spectrum inside which some wavelengths have reduced penetration depths. Recently, using an optical spectrum shifted to the infrared region,^18^ penetration depth was increased by roughly 1 mm, but spatial resolution appears to decrease rapidly with depths greater than 1 mm. OCT has broadened its application field to the imaging of blood vessels.^19^ This later technique, called OCTA, uses the temporal variation of the OCT signal caused mainly by moving red blood cells. OCTA is now a well-established technique in the field of ophthalmology^20^^–^^22^ and seems promising for skin characterization.^23^ Among the numerous modalities that emerged from OCTA, Doppler OCT^24^ can image both blood flow dynamic and tissue structure by combining Doppler velocimetry with OCT. In addition, speckle variance OCT^25^ is sensitive to flow perpendicular to the scanning beam by measuring the intensity speckle change in the OCT signal. Taking into account the vector nature of the electric field, polarization-sensitive OCT (PS-OCT) can detect the birefringent properties of the sample.^26^ However, the interferometric signal of the OCT is subjected to polarization-induced artifacts due to polarization mismatch between the sample and reference beams.^27^ This difficulty has recently been bypassed by a PS-OCT angiography system at the price of a dual-channel OCT setup.^28^

All of these mentioned OCTA techniques inherit the main characteristics of OCT and especially its vulnerability to multiple scattering that is known to be one of the main degrading phenomenon, especially concerning the penetration depth and image contrast.^29^^–^^32^ In OCTA, in addition to projection artifacts^33^ and the fading effect^27^ that have to be compensated for, multiple scattering that impairs the OCT signal is increased because of the high difference of the refractive index between the serum and the cytoplasm of red blood cells. In contrast to such OCT scanning-based techniques, the method that we propose almost exclusively uses multiple scattering to image the vascular flux and thus is expected to access deeper structures for a given wavelength.

Finally, current imaging techniques do not allow for a simple, reproducible, rapid, and noninvasive observation of the subsurface microvascularization for a large field of view. Moreover, high-resolution imaging up to penetration depths of a few millimeters is, until now, not covered by any noninvasive method in strong scattering media. We aim here to fill this gap by introducing a new technique that can be considered to be a polarimetric upgrade of laser speckle imaging experiments such as laser speckle contrast analysis^34^ or laser speckle contrast imaging,^35^ both based on the pioneering work of Fercher and Briers.^36^ These works use the fact that the coherent backscattered light from moving particles leads, when observed with an adequate integration time, to a blurring effect of the detected optical interference called speckle^37^^,^^38^ and thus to a decrease of its temporal or spatial contrast. Previous works from our team^39^^,^^40^ accurately analyzed the polarimetric properties of speckle at the scale of its correlation length. The various polarimetric patterns were found to be very dependent on the scattering origin: whereas surface scattering mainly conserved the illumination state of polarization in the speckle pattern, multiple scattering that occurs generally deeper in volumes did spread the polarimetric states all over the Poincare sphere.^41^ Moreover, polarimetric-dependent variations in the laser contrast imaging signal have been observed recently in studies of a blood vessel mimic flow phantom^42^ and a mouse brain microvascularization.^43^

Starting from these observations, we propose in this work upgrading the LSCI technique using orthogonal polarimetric states (patent FR1750833A) between illumination and detection to mainly suppress the surface scattering contribution and enhance the signal originating from multiple scattering in deeper skin layers. Thus, we propose calling this technique LSOCI for laser speckle orthogonal contrast imaging. We emphasize that, according to this new technique, any fully polarized state of illumination can be used, but the contribution backscattered from the skin has to be projected onto the diametrically opposite polarimetric state in the Poincaré space before detection. We remark that this polarimetric filtering results in the detection of the temporal variation of the spatially depolarized speckle field provoked by multiple scattering. Moreover, we point out that conventional LSCI or similar techniques detect a large part of the first-order scattering, whereas our method aims at removing this contribution to reveal the multiple scattering that occurs in deeper layers in the volume.

We first introduce a simple theoretical model that defines a new microvascular activity index specific to our polarimetric setup. Then, we illustrate experimentally the effect of the polarimetric filtering on a human’s finger and mastoid. We choose these two areas because they exhibit very different microvasculature patterns. Then, we reveal the imaging potentiality of the LSOCI technique on different parts of the body. Finally, we examine the dynamic and stability of the signal.

## 2 Calculation of the Microvascular Activity

We illuminate the skin with fully polarized coherent light in the near-infrared region at the wavelength of 785 nm. A CMOS camera continuously records a stack of raw images, the intensity distribution of which is mainly driven by the coherent sum of the photons backscattered from moving red blood cells. This coherent sum generates a speckle that is driven by the relative phases of the backscattered contributions and thus is extremely sensitive to micromovements of the scatterers. These backscattered contributions contain the surface scattering that conserves mainly the polarization state of the illuminating laser light and the volume scattering, which is composed mainly of multiscattered photons for which the polarimetric states spread rapidly all over the Poincaré sphere with increasing multiscattering.^39^ Thus, after passing through a polarizer in which the eigen axis is set orthogonal to the polarization state of illumination, mainly volume scattering is conserved, and the camera detects, on a pixel indexed by (i,j), the corresponding spatial intensity distribution I(i,j) that fluctuates due to the movements of the scatterers. Thus, for a given integration time, the recorded pattern will be increasingly blurred with a faster movement of red blood cells. To quantify this blurring process, we define in each pixel (i,j), a temporal speckle contrast $C_{⊥}$ along the stack of N sequentially acquired raw images $I_{k},k=1,\ldots ,N$. This contrast characterizes the temporal evolution of the backscattered field after passing through the linear polarizer set orthogonal to the polarization state of the laser illumination:

$$C_{⊥}(i,j)=\frac{\sqrt{\frac{1}{N}\sum_{k=1}^{N}{\left(I_{k}(i,j)-\overset{¯}{I(i,j)}\right)}^{2}}}{\overset{¯}{I(i,j)}},$$ (1)

where $\overset{¯}{I(i,j)}$ is the mean value of the images along time $\overset{¯}{I(i,j)}=\frac{1}{N}\overset{N}{\underset{k=1}{\sum }}I_{k}(i,j)$.

From the theoretical point of view, the speckle contrast is expressed as a function of the normalized autocorrelation function γ(t) of the backscattered field:^44^

$$C^{2}=2\beta \int_{0}^{T}(1-\frac{t}{T}){\left|\gamma (t)\right|}^{2}\frac{dt}{T},$$ (2)

where T is the integration time of the camera and β is a term appearing in Siegert’s relation^45^ that, under the assumption of a Gaussian distribution for the field, relates its normalized autocorrelation function to that of the intensity. β depends on the amount of static scattering present in the sample, the spectral properties of the source, and the detector speckle averaging and noise. Our main concern here is how, in the presence of a dynamic component, the contrast deviates from the theoretical value of 1 obtained in the case of a purely static fully developed speckle. We now address the relationship between the contrast and the correlation times involved in the models of the considered velocity distributions.

In general, Gaussian velocity distribution is assumed to describe a particle flow, whereas Lorentzian distribution is expected to model Brownian movements. The actual distribution is expected to be a combination of both.^46^

As demonstrated in Ref. 47, using the normalized autocorrelation functions $\left|\gamma_{l}(t)|=\exp(-t/\tau_{cl}\right)$ and $\left|\gamma_{g}(t)|=\exp(-\pi t^{2}/2\tau_{cg}^{2}\right)$ for the Lorentzian and Gaussian velocity distributions, respectively, the speckle contrast is expressed as follows:

$$C_{l}^{2}=\frac{\tau_{cl}}{T}+\frac{1}{2}{\left(\frac{\tau_{cl}}{T}\right)}^{2}[e^{-\frac{2T}{\tau_{cl}}}-1],$$ (3)

$$C_{g}^{2}=\frac{\tau_{cg}}{T}\;\mathrm{erf}(\sqrt{\pi }\frac{T}{\tau_{cg}})-\frac{1}{\pi }{\left(\frac{\tau_{cg}}{T}\right)}^{2}(1-e^{-\pi {\left(\frac{T}{\tau_{cg}}\right)}^{2}}),$$ (4)

where $C_{l}$ and $C_{g}$ are the contrast in the Lorentzian and Gaussian hypotheses, respectively, and $\tau_{cl}$ and $\tau_{cg}$ are their corresponding correlation time. Both contrast functions $C_{l}(x)$ and $C_{g}(x)$, for large values of $x=\frac{T}{\tau_{cl}}$ or $x=\frac{T}{\tau_{cg}}$, respectively, converge asymptotically to $\sqrt{\frac{1}{x}}$.

This means that, regardless of the velocity distribution assumption, the contrast function C converges to an asymptote proportional to $\sqrt{\frac{\tau }{T}}$ whenever T, the integration time of the camera, is large compared with the correlation time τ.

This result of convergence also appears in the equation used to define the blood flow index:^48^ $\mathrm{BFI}=1/C^{2}=\frac{T}{\beta \tau_{c}}$. The only difference here is the presence of the β coefficient, which is the sole parameter that depends on the imaging system. To avoid the dependence of this coefficient, the relative index is defined as $\mathrm{rBFI}=\frac{C_{\mathrm{ref}}^{2}}{C_{\text{response}}^{2}}$, where $C_{\mathrm{ref}}$ and $C_{\text{response}}$ are the measured contrasts before and after stimulus, respectively.

In our case, we expect the optical penetration depth of the laser to reach more than 2 mm according to the wavelength used^49^ and due to the polarization filtering process. Moreover, as this later prevents surface scattering, the correlation time of each pixel is mainly driven by multiscattered photons and thus implies volume scattering. Therefore, we define the volume microvascular activity index in each pixel (i,j) as

$$\mathrm{VMAI}(i,j)=\frac{1}{\tau_{cv}(i,j)},$$ (5)

where

$$\tau_{cv}(i,j)=T.C_{⊥}^{2}(i,j).$$ (6)

As the exact calculation of the blood flux would need *a priori* knowledge of the speed distribution of red blood cells that we never have in practical applications,^50^ we determine the terms of microvascular activity to the term of flux. Indeed, the origins of the backscattered contributions are too complex to expect an absolute quantification of the mean speed of red blood cells. However, we can get a stable and reliable parameter related to their distribution speed that takes into account indifferently both longitudinal and transversal flows of red blood cells and Brownian movements. Thus, the measurements performed by the LSOCI technique, such as those of LSCI, can be considered to be semiquantitative. We point out that the definition of this new VMAI is essential because the measurements in clinical applications will differ whether we perform LSCI (mainly driven by surface scattering) or LSOCI (mainly driven by volume scattering). We remark that, in the near-infrared domain, LSCI practiced without any polarizer in the detection arm detects both a surface and a volume contribution, with a major contribution from the surface. However, if the polarizer in the detection arm, in contrast to LSOCI, matches the one of the incident light, the volume contribution is expected to be strongly reduced.^39^ An important fact is that the measured decrease of contrast depends strongly on the polarimetric setup and thus on the origin of the detected backscattered field, which may come mainly from the surface, the volume, or both.

## 3 Experimental Setup

**Fig. 1:**
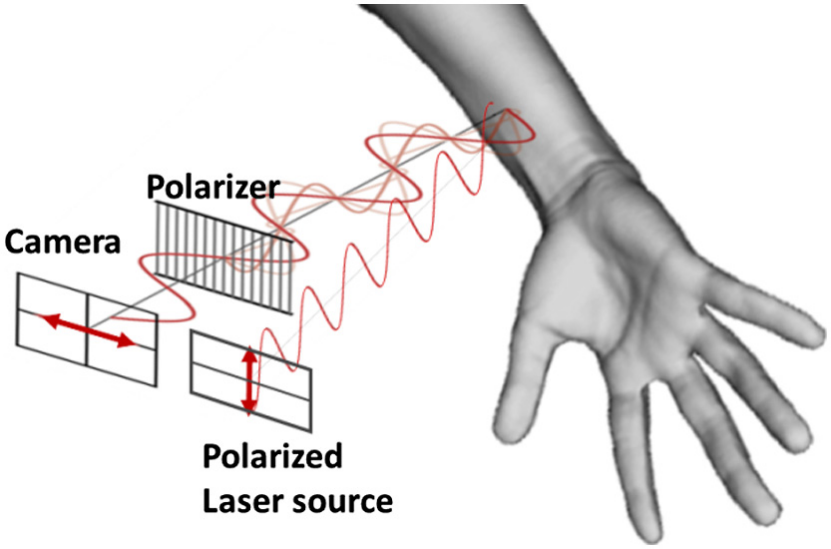
Schematic representation of the polarimetric filtering in the LSOCI technique. Laser illumination is performed in the near-infrared domain, and backscattered photons are sent, before reaching the CMOS camera, through a linear polarizer in which the eigen axis is oriented orthogonal to the laser polarization.

Figure 1 shows a schematic representation of the polarimetric filtering of our LSOCI instrument. A linear polarized TEM00 laser beam (Lambdamini from RGB Photonics GmbH) with a wavelength of 785 nm is sent through diverging optics to illuminate a skin surface of about $12\;{\mathrm{cm}}^{2}$. Backscattered photons are then filtered by a linear grid polarizer (THO-WP25M-UB from Thorlabs) in which the eigen axis is set orthogonal to the polarization state of the laser illumination, before reaching a monochrome CMOS camera of 1.5 million pixels (aca1440220um from Basler).

## 4 Method

We first acquire a stack from 20 to several hundred raw intensity speckle images with an integration time T typically ranging from 10 to 50 ms, from which we deduce for each pixel indexed by (i,j) using Eq. (1) a corresponding temporal contrast $C_{⊥}(i,j)$ that we normalize by the spatial contrast obtained on a static reference sample to take into account the spectral properties of the laser. We then calculate, for each value of this temporal contrast, the VMAI (i,j) in near real time using Eqs. (5) and (6). The result is displayed as the microvascular activity image. When the device works in video mode, a stack from 15 to 20 raw data images is acquired sequentially with an integration time of 10 ms. Each stack is then processed in real time to build each displayed image of microvascular activity. When the device works in acquisition mode, depending on the contrast and the number of fine details requested, we process a stack from 80 to 500 raw data images to build one microvascular activity image. Thus, the acquisition time including the processing of the stack of data varies from less than one second to a few seconds for a conventional PC. In this later acquisition mode, an additional sequential RGB illumination of the skin is synchronized with the acquisition of three more raw data images to build a classical color image corresponding and nearly perfectly stackable to the microvascular activity image. Finally, when we need VMAI measurements, only 20 raw images with integration times of 50 ms were found to provide stable results. In this later mode, longer integration times are preferred to fulfill the large requirement T compared with the decorrelation time $\tau_{cv}$.

## 5 Results

### 5.1 Effects of the Polarimetric Filtering

Until now, linear polarizers orthogonal to the incident polarimetric state of the illuminating laser light had been used in a few imaging systems to suppress the specular component and to prevent the dazzling of the camera. However, we would like to emphasize that such an orthogonal illumination/detection scheme not only suppresses the specular component but tends to suppress all of the first-order scattering that corresponds generally to the surface contribution. This is especially the case if we consider the skin that backscatters distinct polarimetric patterns between its surface and its volume in the near-infrared domain. Thus, such polarimetric filtering has the effect of strongly decreasing the response of the surface, to the benefit of multiscattering generated in the volume, in the deepest layers of the skin.

**Fig. 2:**
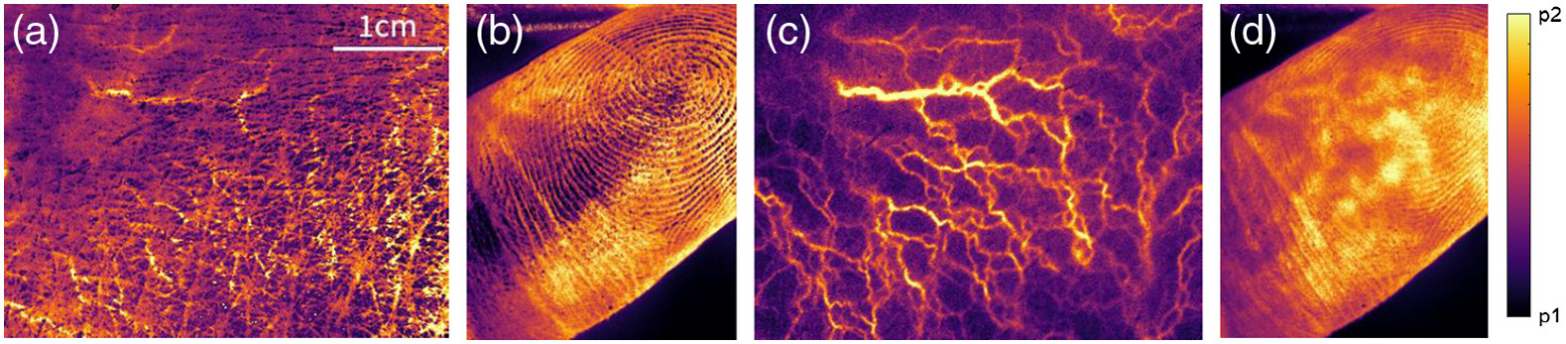
Effect of the polarimetric filtering. Observation of a mastoid and a finger without any polarimetric filtering in (a) and (b), respectively, and with the LSOCI method in (c) and (d), respectively. Figures (a) and (b) show only superficial structures, whereas (c) and (d) reveal the microvascular activity pattern deeper into the skin. $p_{1}$ and $p_{2}$ are the threshold values for the image display, automatically calculated from the 1st and 99th percentiles of the intensity distribution.

We observe experimentally the effect of the polarimetric filtering allowing for the acquisition of the microvascular activity image as defined in the Sec. 2. In Figs. 2(a) and 2(b), we show the images of a mastoid and finger generated from a nonfiltered dynamic optical speckle respectively. Figures 2(c) and 2(d) show the same places of the body but obtained with the LSOCI technique, implying that the surface backscattered contribution is filtered out. We observe first that, whereas Fig. 2(a) exhibits fine details of the skin surface of the mastoid with numerous stripes, the corresponding LSOCI image Fig. 2(c) reveals the underlying microvascular pattern and completely removes the stripes. We observe a similar behavior on a finger between Figs. 2(b) and 2(d): fingerprints are visible in Fig. 2(b), whereas they mostly disappear in Fig. 2(d). This later reveals a complex structure of anastomotic microvascular activity hot spots typical of the numerous observations that we have performed on fingers.

### 5.2 Examples of Skin Microvasculature Observations

**Fig. 3:**
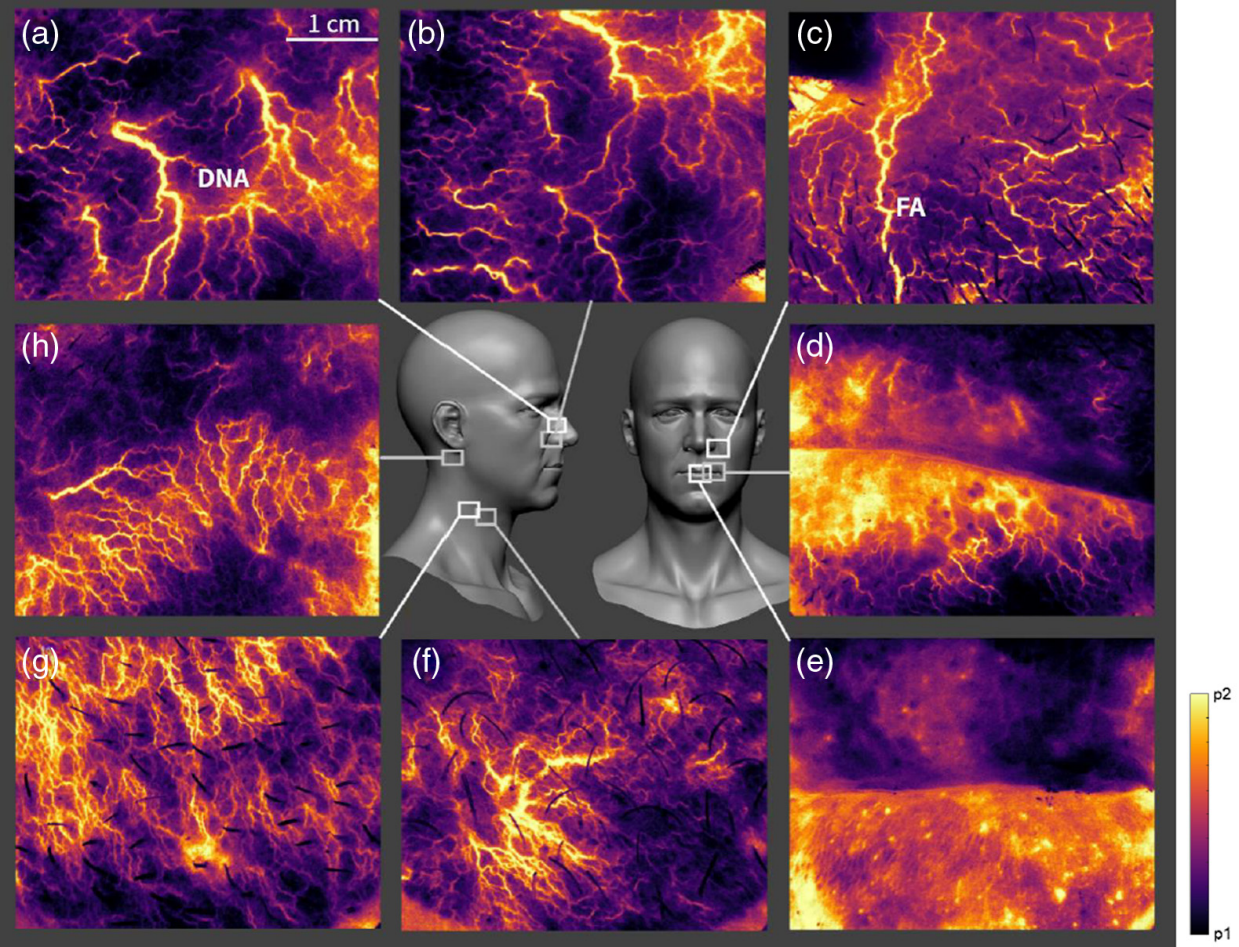
Examples of *in vivo* microvascular activity acquisitions. Images are obtained with the LSOCI technique (a)–(e) on different parts of the human face; (f), (g) on the neck; and (h) on the mastoid. p1 and p2 are the threshold values for the image display, automatically calculated from the 1st and 99th percentiles of the intensity distribution. The dorsal nasal artery (DNA) and the facial artery (FA) can be observed respectively in (a) and (c).

Our LSOCI instrument can reveal near-instant *in vivo* and high-resolution images of the transcutaneous microvasculature. To support this point, we give in Fig. 3 a set of typical images observed on the neck and over the head that have been shown to exhibit particularly fine microvasculature patterns. For each microvascular activity image, 500 raw images acquired with an integration time of 10 ms were processed as described in Sec. 4. We can observe three main sorts of structures: fine and intense microvessels that exhibit various thicknesses; hot spots of various dimensions and shapes observed mainly on the lips in Figs. 3(d) and 3(e); and diffuse areas of lower intensity levels appearing in violet that, in our experience, have shown to be nearly as stable as the other structures on repeating acquisitions. These diffuse areas could be composed of microvessels not optically resolved by our system. We remark that each part of the body corresponds to a typical pattern. Figures 3(a)–3(c) show typical microvessels structures observed near the nose. We can recognize the dorsal nasal artery in Fig. 3(a) and the facial artery in Fig. 3(c). We note that the facial artery is known to be at least 2.5 mm deep in this region.^51^ Acquisitions Figs. 3(d) and 3(e) performed on the lips show both microvessels and hot spots probably related to the coronary nature of labial microvascularization. We observe a higher activity on the lower lip. Figures 3(f)–3(h) performed on the mastoid and the neck show numerous crossing microvessels.

To perform real-time monitoring, it is possible to work with a reduced quantity of raw data to build the microvascular activity images. A setup with only 20 raw images acquired to build the microvascular pattern provides a weaker but sufficient signal-to-noise ratio using a typical integration time of 10 ms. Thus, a refresh rate near 7 Hz can be reached, and it becomes possible to slowly scan the skin by translating the instrument’s head at its surface. We provide an extra file that shows exactly what the hospital practitioner could see in real time when working in this video mode to scan a human face (see Fig. 6). It is possible to increase the quality of the microvascular activity image at the expense of the refresh rate.

**Fig. 4:**
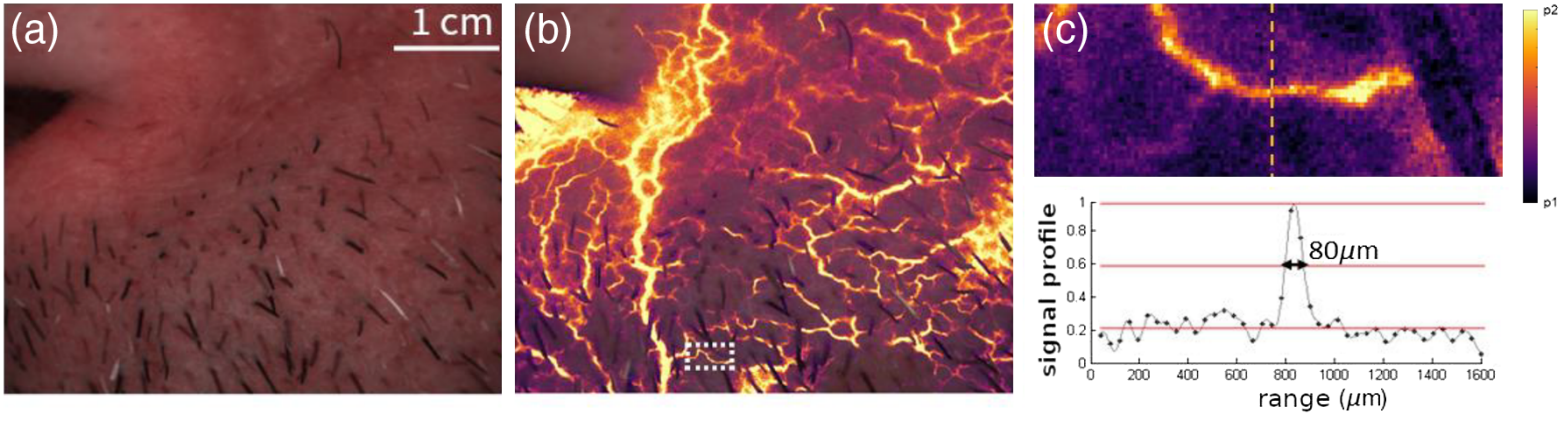
Evaluation of the spatial resolution of the LSOCI instrument. (a) Example of color image near the nose for which we overlay the microvascular activity image in (b). (c) A cross section analysis of a fine vessel squared in (b) exhibits a full width at half maximum of 80  μm.

Our LSOCI instrument allows for a near simultaneous recording of the classic color image that we can superimpose on the microvascular activity image. This overlay would be particularly useful, for example, for skin cancer monitoring. Indeed, skin tumors such as melanoma and those from nonmelanoma skin cancers are known to be abnormally vascularized.^52^^,^^53^ Physicians detect them usually by their shape, colors, and evolution in time. Thus, the classical color image and the microvascular activity image, which is able to reveal the neoangiogenesis, bring complementary information. We give an example of such an overlay on healthy skin in Fig. 4.

We show in Fig. 4(a) a classical color image and in Fig. 4(b) the corresponding microvascular activity image that was overlaid on Fig. 4(a). To assess the spatial fineness of the measurement, we selected a thin microvessel framed in Fig. 4(b) and magnified in Fig. 4(c). We calculated the full width at half maximum of the response in the signal cross-section. Since the spatial sampling of our setup was set to 40  μm/pixel, we estimate the width of the structure visible in Fig. 4(c) to be 80  μm. This value, termed resolution here, represents the smallest attainable dimension of the observed structure with our setup.

**Fig. 5:**
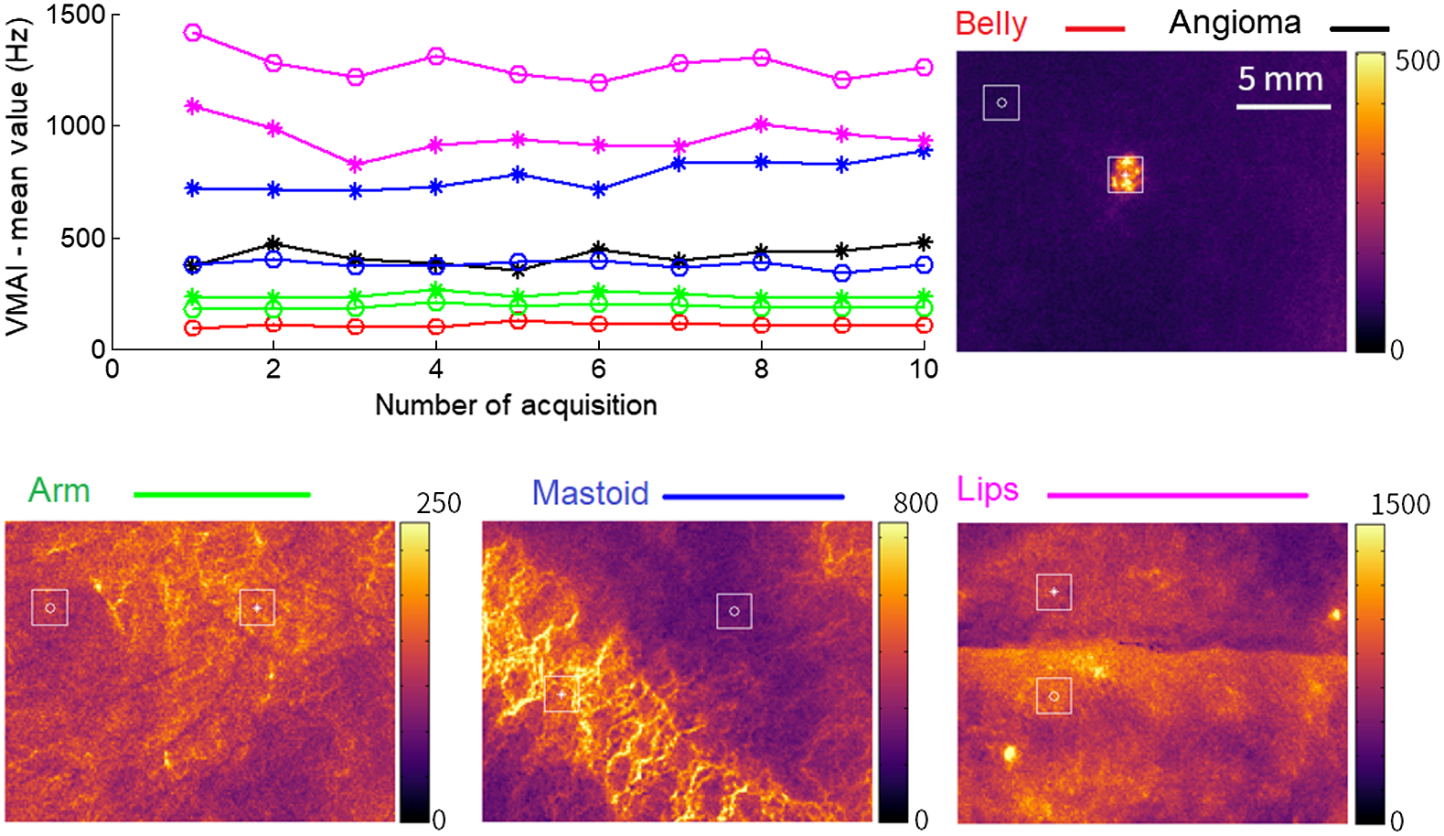
Evaluation of the stability of the VMAI. Examples of values extracted from squared regions of interest distributed on different parts of the body. For each region, we performed 10 redundant acquisitions. The VMAI exhibits a signal dynamic ranging from 99 to $1414\;s^{-1}$, with a mean relative standard deviation of 7%. The lips exhibit the highest microvascular activity, as expected.

To evaluate the ability of the LSOCI technique to quantify the microvascular activity *in vivo*, we show in Fig. 5 measurements on different parts of the body including the arm, the belly that exhibits a cherry angioma, the mastoid, and the lips. We show a typical example of 10 redundant acquisitions on each zone to evaluate the stability of the signal. At this stage, we measure only one upper limit of the standard deviation related to the stability of our optical setup, since this standard deviation also includes natural biological fluctuations. According to Sec. 2, integration time was set to 50 ms to ensure that the condition T was strongly superior to the decorrelation time $\tau_{\mathrm{cv}}$, and 20 raw images were used to build one microvascular activity image. Thus, the sum of the integration times of all of the raw images used to build one microvascular activity image is 1 s. It is five times less than the total integration time used to obtain images shown in Fig. 3. As a result, a much lower signal-to-noise ratio is obtained, but it is found to be sufficient for reaching a stable quantification of the signal. Comparing the different parts of the body, the VMAI as defined in Sec. 2 exhibits a dynamic ranging from 99 to $1414\;s^{-1}$, with the lower lip showing the highest mean value ($1268.6\pm 65.2\;s^{-1}$) as expected. We obtained the lowest mean values for the belly ($111.3\pm 9.3\;s^{-1}$). The relative standard deviations lie between 4.7% for the mastoid and 9.7% for the angioma, with a mean of 7%. In our experience, a part of the variations of the VMAI observed in Fig. 5 comes from the state of the cardiac cycle at the beginning of the measurement. An interesting consequence is that the cardiac cycle could be observed from a simple angioma. The dynamic and relative stability of these measurements demonstrate the possibility of performing semiquantitative measurements as discussed in Sec. 2.

## 6 Discussion

The proposed imaging technique is based on the detection of spatially depolarized speckle fields generated from multiple scattering into red blood cells. It reveals new images of the human skin microvascularization and exhibits promising observation depth capabilities and spatial resolution. This latter is shown to reach 80  μm in our system and can appear surprisingly fine, considering the unavoidable movement of both the practitioner and the patient during signal acquisition. We explain this unexpected fine resolution as a result of a weak contact between the circular head of our instrument and the skin around the observed area that avoids relative movements between them. We would like to emphasize that our signal benefits from an interesting penetration depth due to not only the wavelength used but also the selective detection of multiscattered photons coming from deeper skin layers. It is interesting to remark that, in the case of OCT, by contrast, multiple scattering limits the observation depth. A typical vascular structure that is known to be deeper than 2.5 mm has been observed on the face through the skin. However, an accurate determination of the maximum penetration depth of LSOCI for human skin should be investigated further. We believe that the proposed technique could bridge the gap between high spatial resolution imaging techniques such as confocal microscopy (CM) and OCT that are typically limited to a submillimeter depth and other imaging techniques such as MRA, CTA, and noninvasive photoacoustic imaging that exhibit deep penetration but with a lower spatial resolution. Moreover, we would like to point out that the nature of the observable physical parameter in our device is different and thus complementary to the one of CM and OCT. The signal of the LSOCI technique is based on the decorrelation time of the speckle intensity distribution traducing movements of red blood cells and can be interpreted as a volume microvascular activity. Thus, the LSOCI signal has benefits, such as that of the PET scan^54^ regarding tumor metabolism, of a real-time activity evaluation, but it is not aimed at imaging the structure of tissues such as OCT and CM. We underline that, as with all dynamical speckle imaging techniques, our signal, generated by optical interference, is naturally extremely sensitive to submicromovements and is expected to be promising for very early skin cancer detection by imaging the early neoangiogenesis.

To conclude, we have proposed the LSOCI technique that uses the polarimetric properties of the dynamic speckle backscattered from moving red blood cells. A polarimetric filtering prevents the contribution of the skin surface from reaching the camera and thus favors the detection of multiscattered photons coming from the volume in deeper skin layers. We have introduced a new microvascular activity index named VMAI that is specific to the polarimetric setup of LSOCI. This later has shown to exhibit, on different parts of the body, a mean stability of 7% in spite of the cardiac cycle effect. In addition to these quantitative measurements, we have demonstrated the possibility of observing the skin microvascularization in real time, revealing structures as small as 80  μm. LSOCI is a simple full field and noninvasive technique that is eye safe and low cost. The short acquisition time below the second and contact mode allow for minimizing the proclivity for image artifacts due to patient movement. With its large field of view and penetration depth capabilities, we believe that LSOCI can lead to an interesting clinical tool for easy and rapid assessment of various skin microvascular pathologies that need a deeper observation than OCTA without requiring its depth-resolved imaging capabilities. Further upgrades of the method could include a dual-polarization detection setup that would detect the backscattered contributions simultaneously parallel and orthogonal to the illumination polarization to distinguish between surface and deeper vascularization. We expect numerous clinical applications concerning the pathologies involving microvascularization, such as the monitoring and quantification of inflammatory and degenerative skin diseases, including the detection and follow up of skin cancers. Facial plastic surgery and immediate post-surgery follow-up of flaps and grafts could also be addressed. In future works, close collaboration with physicians will be necessary to identify the link between the LSOCI signal and the various skin pathologies.

## 7 Appendix: Supplemental Material

**Fig. 6:**
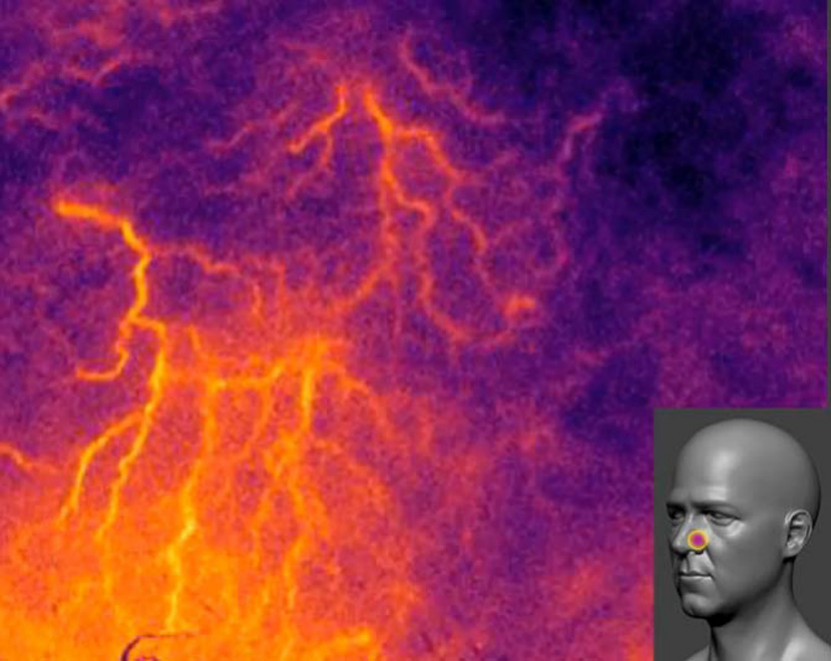
Microvascular activity, as can be viewed by an operator in real time (Video 1, MPEG, 12.5 MB [URL: https://doi.org/10.1117/1.JBO.27.4.046003.1]).

Video 1 shows the trajectory followed by the TMV head over a human face with the corresponding images of microvascular activity, as can be viewed by an operator in real time (Fig. 6).

## Code, Data, and Materials Availability

Data underlying the results presented in this paper are not publicly available at this time but may be obtained from the authors upon reasonable request.
